# An Active Contour Model for the Segmentation of Images with Intensity Inhomogeneities and Bias Field Estimation

**DOI:** 10.1371/journal.pone.0120399

**Published:** 2015-04-02

**Authors:** Chencheng Huang, Li Zeng

**Affiliations:** 1 Key Laboratory of Optoelectronic Technology and System of the Education Ministry of China, Chongqing University, Chongqing, 400044, China; 2 College of Mathematics and Statistics, Chongqing University, Chongqing, 401331, China; 3 Engineering Research Center of Industrial Computed Tomography Nondestructive Testing of the Education Ministry of China, Chongqing University, Chongqing, 400044, China; Hong Kong University of Science and Technology, HONG KONG

## Abstract

Intensity inhomogeneity causes many difficulties in image segmentation and the understanding of magnetic resonance (MR) images. Bias correction is an important method for addressing the intensity inhomogeneity of MR images before quantitative analysis. In this paper, a modified model is developed for segmenting images with intensity inhomogeneity and estimating the bias field simultaneously. In the modified model, a clustering criterion energy function is defined by considering the difference between the measured image and estimated image in local region. By using this difference in local region, the modified method can obtain accurate segmentation results and an accurate estimation of the bias field. The energy function is incorporated into a level set formulation with a level set regularization term, and the energy minimization is conducted by a level set evolution process. The proposed model first appeared as a two-phase model and then extended to a multi-phase one. The experimental results demonstrate the advantages of our model in terms of accuracy and insensitivity to the location of the initial contours. In particular, our method has been applied to various synthetic and real images with desirable results.

## Introduction

Image segmentation still plays an important role in image understanding and computer vision. Active contour models (ACMs) have been widely applied to image segmentation since their introduction [1]. ACMs can obtain closed object contours as segmentation results, which can be conveniently used for shape analysis and recognition. The active contours can utilize various types of prior knowledge, such as image intensity distribution information, boundary shape information, and texture information [2–4], to obtain accurate results for object boundaries in image analysis.

ACMs can be categorized as edge-based models [5–8] or region-based models [9–15]. Edge-based models often use an image gradient to force the active contours to move toward the desired object’s boundaries. These models are typically sensitive to noise, and weak boundaries, which have small gradient values, may cause edge leakage. Region-based models use image statistical information to attract the active contours to the object boundaries. They outperform edge-based models in many cases, such as computer tomography (CT) and magnetic resonance (MR) images. However, traditional region-based models rely on the idea that the intensity of images is homogeneous, which is not suitable for images with intensity inhomogeneity. For example, Chan and Vese proposed the Chan-Vese (CV) model [10], or piecewise constant (PC) model, under the assumption that an image consists of two statistically homogeneous regions and has a distinct mean pixel intensity in each region.

Intensity inhomogeneities often occur in real images, such as CT and MR images. Jungke et al. [16] illustrated that the most important problem for brain MR image segmentation is the occurrence of intensity inhomogeneities. Spatial intensity inhomogeneity are generally related to the properties of the MR image device and include static field inhomogeneity, bandwidth filtering of the data, eddy currents driven by field gradients, and especially radio frequency (RF) transmission and reception inhomogeneity [17]. The spatial intensity inhomogeneity caused many difficulties for MR image segmentation. Many research applications have been used for images with intensity inhomogeneity [2, 4, 18–22]. However, these methods do not consider the correction of the bias field, which is critical for particular clinical diagnoses. Many bias field correction methods have been widely studied in recent years [23–26]. The most popular methods for bias field estimation are based on image segmentation [27–30]. In these methods, the bias field estimation and segmentation are performed simultaneously in each iteration to obtain a final optimal solution. To solve the segmentation problem of intensity inhomogeneity and estimate the bias field, Li et al. [29] proposed a novel variational level set model, that uses the weighted K-means clustering method to evaluate the bias field of image intensities in a neighborhood around each point in the image domain. By considering the retinex model [31], Li’s model can obtain accurate segmentation results and bias field estimates in the presence of intensity inhomogeneity, such as in MR and camera images. Some related methods, which have capabilities similar to Li’s model in terms of considering intensity inhomogeneity, have been proposed [2, 18, 30]. Zhang et al. proposed a locally statistical active contour model (LSACM) to segment images with intensity inhomogeneity and bias correction [32], which has good performance with bias correction. However, these models are sensitive to the location of the initial contours [33].

In this paper, we introduce the local regional difference to Li’s model. With this regional difference, the modified model can improve both the accuracy of the segmentation results for images with intensity inhomogeneity and the estimation of the bias field. We define a new local clustering criterion by collecting the local region difference in the entire image domain. Under this local clustering criterion, the bias field and segmentation results can be effectively corrected in each iteration for different initial contours. Experiments demonstrate that our model can obtain more accurate results.

The remainder of this paper is organized as follows. In the next section, we review some well-known region-based models and their limitations and then present our modified model and the numerical algorithm. The Results and Discussions section presents and discusses the experimental results. Finally, the Conclusions sections presents the conclusions of this study.

## Models

### The piecewise constant (PC) model

**Fig 1:**
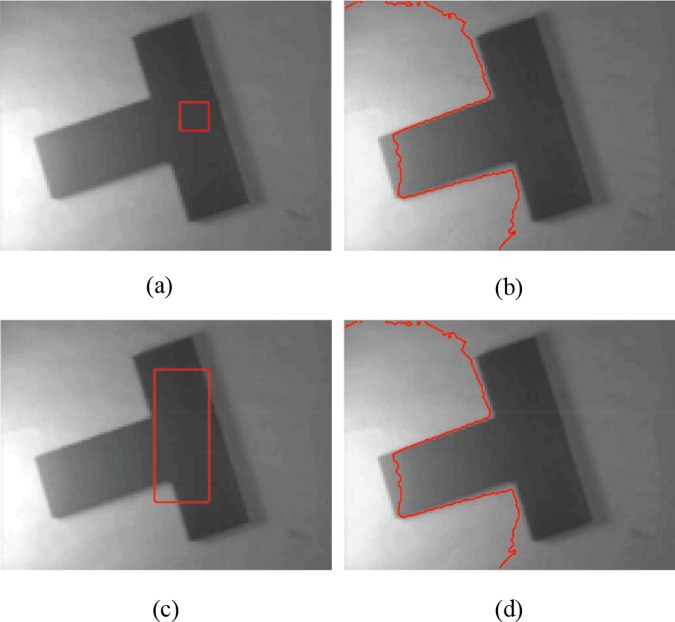
Segmentation of a real T-shaped image with intensity inhomogeneity by CV model. (a), (c) The original image with red initial contours. (b), (d) Segmentation results of the CV model.

Chan and Vese proposed the CV model [10] to solve the segmentation of two-phase images whose mean intensities can be distinct. The main concept of the CV model is to search for a particular partition of a given image *I*(**x**) into two regions, one representing the objects to be detected and the other representing the background. For the given image *I*(**x**), they proposed to minimize the following energy functional [10]

$$\begin{array}{c} E^{CV}\left(C,c_{1},c_{2}\right)=\lambda_{1}\int_{in(C)}|I\left(x\right)-c_{1}{|}^{2}dx+\lambda_{2}\int_{out(C)}|I\left(x\right)-c_{2}{|}^{2}dx+\nu \left|C\right|,\;x\in \Omega . \end{array}$$ (1)

where *λ*_1_ and *λ*_2_ are positive constants, *ν* ≥ 0, *in*(*C*) and *out*(*C*) represent the inner and outer regions of the contour *C*, respectively, and *c*_1_ and *c*_2_ are two constants representing the mean image intensities in *in*(*C*) and *out*(*C*), respectively, Thus, the CV model is also called the piecewise constant (PC) model. The first and second terms of (1) are the inner and outer data fidelity terms of the contour *C*, respectively. The third term of (1) is the length term, which is used to regularize the contour *C*. The CV model performs well in image segmentation due to its ability to detect objects whose boundaries are either smooth or not necessarily defined by a gradient and to obtain a larger convergence range; moreover, it is less sensitive to the initialization. However, when the intensities inside or outside of the curve *C* are not homogeneous, the constants *c*_1_ and *c*_2_ may not accurately describe the variance in the local region; thus, the CV model may fail to segment images with intensity inhomogeneity (Fig. 1(b), (d)).

### Li’s model

Li et al. [29] considered the bias field in the local region to segment an image with intensity inhomogeneity and estimate the bias field. Based on the retinex theory [31], the considered bias field model can be written as follows:

$$\begin{array}{c} I=b\cdot J+n \end{array}$$ (2)

where *I* is an image to be measured, *b* is the bias field, *J* is the true image and *n* is the additive noise. The assumptions for the true image *J* and bias field *b* are as follows:

(*A*1) The bias field *b* varies slowly over the entire image domain.
(*A*2) The true image intensities *J* are approximately constant within each class of issue, i.e., *J*(**x**) ≈ *c*_*i*_ for **x** ∈ Ω_*i*_, where ${\left\{\Omega \right\}}_{i=1}^{N}$ is a partition of Ω.

Let Ω_**x**_ = {**y**:∣**y**−**x**∣ ≤ *ρ*} be the neighborhood of **x** in the image domain with a small radius *ρ* and Ω_**x**_∩Ω_*i*_ represent the partition of Ω_**x**_ produced by the i-th partition Ω_*i*_ of the image. Based on assumption (*A*1), the value *b*(**y**) for all Ω_**x**_ can be close to *b*(**x**); then, in the small region Ω_**x**_∩Ω_*i*_, the product of the bias field *b*(**y**) and image intensity *J*(**y**) can be an approximated by *b*(**y**)*I*(**x**) ≈ *b*(**x**)*c*_*i*_ according to assumption (*A*2). By using the K-means clustering method, they considered all the *N* partitions of image, and defined a local energy function as follows:

$$\begin{array}{c} E_{x}=\sum_{i=1}^{N}\int_{O_{x}\cap \Omega_{i}}K_{\sigma }\left(x-y\right){\left|I\left(y\right)-b\left(x\right)c_{i}\right|}^{2}dy \end{array}$$ (3)

where *K*_*σ*_(*s*) is a weighted function, which can be expressed as a Gaussian kernel function with standard deviation *σ*:

$$\begin{array}{c} K_{\sigma }\left(s\right)=\left\{\begin{array}{cc} \frac{1}{\sqrt{2\pi }\sigma }e^{-{\left|s\right|}^{2}/2\sigma^{2}}, & |s|<\rho \\ 0, & otherwise \end{array}\right. \end{array}$$ (4)

To find an optimal of the entire image domain Ω, an overall energy for all **x** is defined as

$$\begin{array}{c} E=\int \left(\sum_{i=1}^{N}\int_{\Omega_{i}}K_{\sigma }\left(x-y\right){\left|I\left(y\right)-b\left(x\right)c_{i}\right|}^{2}dy\right)dx \end{array}$$

When considering the case in which *N* = 2 in Li’s model, after introducing the level set function *ϕ*(**x**), the overall energy can be written as

$$\begin{array}{c} E=\int \left(\sum_{i=1}^{2}\int_{\Omega }K_{\sigma }\left(x-y\right){\left|I\left(y\right)-b\left(x\right)c_{i}\right|}^{2}dy\right)u_{i}\left(\phi \left(x\right)\right)dx \end{array}$$

where *u*_1_(*ϕ*(**x**)) and *u*_2_(*ϕ*(**x**)) are the membership functions of each cluster, *u*_1_(*s*) = *H*(*s*), and *H*(*s*) is the heaviside function defined as $H(s)=\frac{1}{2}[1+\frac{2}{\pi }\arctan(\frac{s}{\varepsilon })]$ (*ε* > 0), *u*_2_(*s*) = 1−*H*(*s*). For fixed *ϕ*, *c*_1_ and *c*_2_, the optimal bias field *b* can be computed by minimizing the local energy *E*_**x**_ in (3) as follows:

$$\begin{array}{c} b=\frac{K_{\sigma }*\left(\sum_{i=1}^{2}c_{i}u_{i}\left(\phi \right)\right)}{K_{\sigma }*\left(\sum_{i=1}^{2}c_{i}^{2}u_{i}\left(\phi \right)\right)} \end{array}$$ (5)

where ‘∗’ denotes the convolution operation. Similarly, the optimal *c*_1_ and *c*_2_ can be computed by

$$\begin{array}{c} c_{i}=\frac{\int \left(K_{\sigma }*b\right)Iu_{i}\left(\phi \right)dx}{\int \left(K_{\sigma }*b^{2}\right)u_{i}\left(\phi \right)dx},\;i=1,2 \end{array}$$ (6)

**Fig 2:**
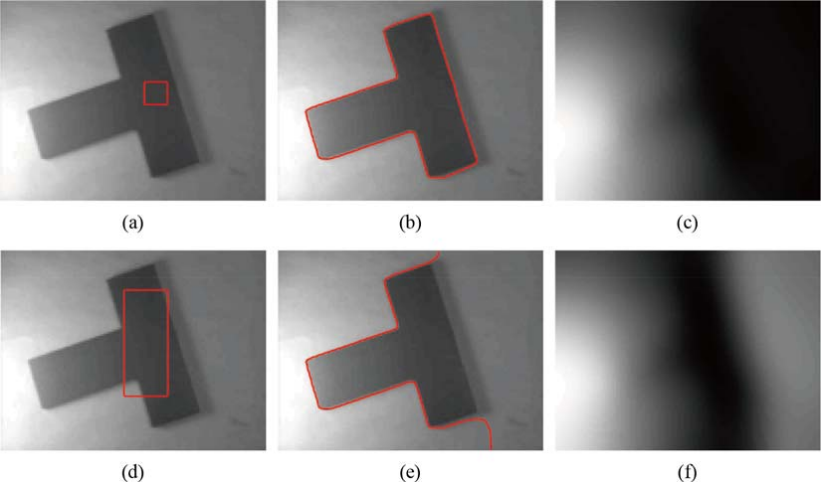
Segmentation of Li’s model. (a), (d) The original image with red initial contours. (b), (e) Segmentation results of Li’s model. (c), (f) The bias field estimation of Li’s model.

**Fig 3:**
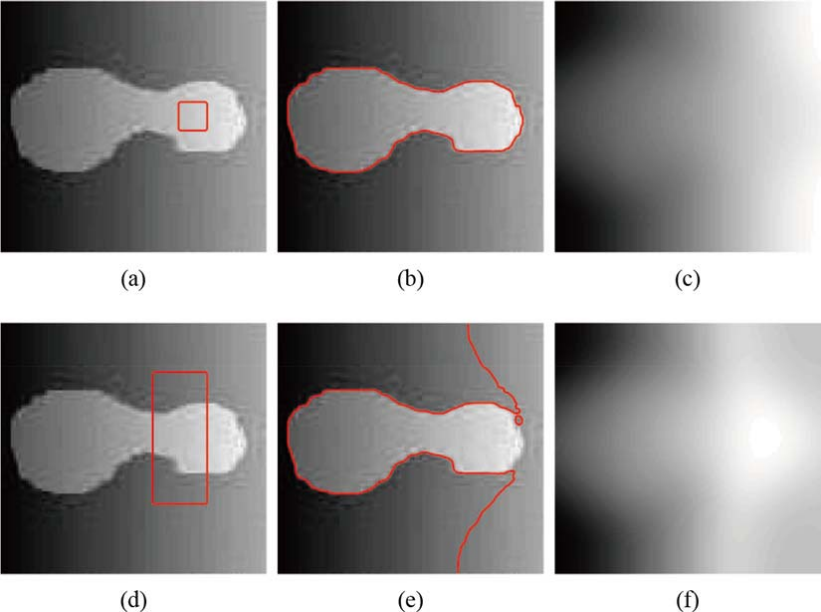
Segmentation of Li’s model. (a), (d) The original image with red initial contours. (b), (e) The final segmentation results of Li’s model. (c), (f) The bias field estimation of Li’s model.

In this case, the image domain Ω is divided into two regions, Ω_1_ = {*ϕ* > 0} (objects) and Ω_2_ = {*ϕ* < 0} (background). Because the local image intensity information is embedded into the energy function, Li’s method can address some types of images with intensity inhomogeneity; however, it still has inherent drawbacks. From (5) and (6), the intensity means *c*_*i*_ (*i* = 1, 2) and bias field *b* are related; thus, the estimation of *c*_1_ and *c*_2_ are critical for obtaining a better estimation of bias field *b*. However, when the object intensity is close to the background in the local region, the estimation of the bias field may be inaccurate, and thus, estimating the bias field using only the mean intensity in the local region is not sufficient. As shown in Fig. 2, Li’s method can obtain the correct segmentation (Fig. 2(b)) when the initial contour is located in the inner part of the object (Fig. 2(a)). However, when the initial contours contain both object and background (Fig. 2(d)), Li’s method fails to segment the object (Fig. 2(e)), and the false segmentation result leads to worse estimation of the bias field (Fig. 2(f)). Similar results can also be seen in Fig. 3. In other words, Li’s method may drop into local minimums [34] and is sensitive to the location of the initial contour; thus, the segmentation results and bias field estimation may be inaccurate in some cases.

### Our model

The segmentation result may affect the bias field correction, and using only the local region intensity means and bias field in Li’s model is not sufficient to approximate the measured image well. Thus, motivated by the contributions and methods of [27–30], we present a new ACM to segment images with intensity inhomogeneity and estimate the bias field, and this model incorporates the local difference information between the measured image and Li’s estimate. In Li’s model, the segmentation result is essential for the estimation of the true image *J* and the bias field; an accurate segmentation result can accurately estimate the bias field, whereas a bad segmentation result cannot do so. In our model, we introduce the difference in the local region of the image domain to improve the accuracy of the segmentation result and bias field estimation. For an input image *I*, the model can be described as follows:

$$\begin{array}{c} I=b\cdot J+d+n \end{array}$$ (7)

where *d* is the difference between the measured image *I* and approximated model *b*⋅*J* in the local region. According to this model, a K-means clustering-based local energy function of our model is defined as follows:

$$\begin{array}{c} E_{x}=\sum_{i=1}^{N}\int_{O_{x}\cap \Omega_{i}}K_{\sigma }\left(y-x\right){\left|I\left(y\right)-b\left(x\right)c_{i}-d\left(x\right)\right|}^{2}dy \end{array}$$ (8)

where *K*_*σ*_(*s*) is the Gaussian kernel function with standard deviation *σ*. By introducing the level set function *ϕ*(**x**) and considering all pixels in image domain, the overall energy can be written as

$$\begin{array}{c} E\left(b\left(x\right),c,d\left(x\right),\phi \left(x\right)\right)=\int \left(\sum_{i=1}^{N}\int_{\Omega }K_{\sigma }\left(x-y\right){\left|I\left(y\right)-b\left(x\right)c_{i}-d\left(x\right)\right|}^{2}dy\right)u_{i}\left(\phi \left(x\right)\right)dx \end{array}$$

where *u*_*i*_ is the membership function and **c** = (*c*_1_, *c*_2_, …, *c*_*N*_). After fixing *σ*, **c** and *d*, we find an optimal bias field *b* that minimizes *E*_**x**_ in (8):

$$\begin{array}{c} b=\frac{K_{\sigma }*\left(\left(I-d\right)\cdot J^{\left(1\right)}\right)}{K_{\sigma }*J^{\left(2\right)}} \end{array}$$ (9)

where ‘∗’ is the convolution operation, $J^{\left(1\right)}=\sum_{i=1}^{N}c_{i}u_{i}(\phi )$ and $J^{\left(2\right)}=\sum_{i=1}^{N}c_{i}^{2}u_{i}(\phi )$, *u*_*i*_(*ϕ*) is the membership function of the partition Ω_*i*_. Similarly, the optimal *c* and *d* can be obtained from (8):

$$\begin{array}{cc} c_{i} & =\frac{\int \left(K_{\sigma }*b\right)\left(I-d\right)u_{i}\left(\phi \right)dx}{\int \left(K_{\sigma }*b^{2}\right)u_{i}\left(\phi \right)dx},\;i=1,...,N\\ d & =\frac{K_{\sigma }*M^{\left(1\right)}}{K_{\sigma }*M^{\left(2\right)}} \end{array}$$ (10)

where $M^{\left(1\right)}=\sum_{i=1}^{N}\left(Iu_{i}(\phi )-bc_{i}u_{i}(\phi )\right)$ and $M^{\left(2\right)}=\sum_{i=1}^{N}u_{i}(\phi )$.

In the process of curve evolving (the zero level set function), we keep the level set function as an approximate signed distance function, especially in the neighborhood of the zero level set [35]. For a general level set function method, the level set function is often computed as a signed distance function and must re-initialize the level set function after some evolution steps, but the re-initialization step is time-consuming and not effective. Li et al. proposed a regularization term [36] as a penalty term to eliminate the re-initialization step. The regularization term can be written as follows:

$$\begin{array}{c} P\left(\phi \right)=\frac{1}{2}\int_{\Omega }{\left(|\nabla \phi \left(x\right)|-1\right)}^{2}dx \end{array}$$

This regularization term can force the level set function to be closed to a signed distance function in the process of curve evolution. We also choose the length term in the CV model for more accurate computation, so a regularization term can be written as

$$\begin{array}{c} E^{R}\left(\phi \right)=\alpha \int_{\Omega }\frac{1}{2}{\left(|\nabla \phi \left(x\right)|-1\right)}^{2}dx+\beta \int_{\Omega }\left|\nabla H\left(\phi \left(x\right)\right)\right|dx \end{array}$$

The final energy functional can be written as follow

$$\begin{array}{c} E^{FEF}\left(b,c,d,\phi \right)=E\left(b,c,d,\phi \right)+E^{R}\left(\phi \right) \end{array}$$ (11)

### The level set variation formulation of our model

In level set methods, the evolving contour (object contours) is represented by zero level set function *ϕ* = 0 in the level set formulation. The case of *N* = 2 and *N* > 2 in the energy represents two-phase and multi-phase formulations, respectively. In the following subsections, we will consider two-phase and multi-phase cases.

#### Two-phase level set variation formulation

For two-phase (N = 2) images, which the whole image domain Ω contains foreground and background. The energy functional can be written as follows:

$$\begin{array}{c} E\left(b,c,d,\phi \right)=\int \left(\sum_{i=1}^{2}\int K_{\sigma }\left(y-x\right){\left|I\left(y\right)-b\left(x\right)c_{i}-d\left(x\right)\right|}^{2}u_{i}\left(\phi \right)dy\right)dx+E^{R}\left(\phi \right) \end{array}$$ (12)

where *u*_1_ and *u*_2_ are the membership functions of Ω_1_ and Ω_2_, *b*, *c*_1_, *c*_2_ and *d* are given as follows:

$$\begin{array}{cc} b & =\frac{K_{\sigma }*\left(\left(I-d\right)\cdot \sum_{i=1}^{2}c_{i}u_{i}\left(\phi \right)\right)}{K_{\sigma }*\sum_{i=1}^{2}c_{i}^{2}u_{i}\left(\phi \right)}\\ c_{i} & =\frac{\int \left(K_{\sigma }*b\right)\left(I-d\right)u_{i}\left(\phi \right)dx}{\int \left(K_{\sigma }*b^{2}\right)u_{i}\left(\phi \right)dx},\;i=1,2\\ d & =\frac{K_{\sigma }*\sum_{i=1}^{2}\left(\left(I-bc_{i}\right)u_{i}\left(\phi \right)\right)}{K_{\sigma }*\sum_{i=1}^{2}u_{i}\left(\phi \right)} \end{array}$$ (13)

**Note 1**: The difference *d* is a matrix with dimension *M* × *N* ([M, N]=size(I)). If the local difference matrix *d* = 0, our method is similar to Li’s method in [29]; however, there are still differences even for an accurate approximating evaluation of the measured image. Thus, for *d* ≠ 0, the local difference variable between image *I*(**x**) and the approximate form *b*(**y**)*c*_*i*_ in each Ω_**x**_∩Ω_*i*_ can be corrected in our model. If there is a large *d* in the local region of **x**, the measured image *I*(**x**) is not well approximated by the local bias field and local piecewise constant, and the active contours will move to the regions that reduce *d*. Thus, the proposed method will obtain more accurate results in each iteration because the local difference in each clustering of the image is considered.

**Note 2**: Wherever the region’s initial contours are located, the difference *d* can always be collected in each local region and corrected by the estimate *b* and *c*_*i*_ to the smaller values between the measured image and approximate *bc*_*i*_, which makes our model insensitive to the initialization of the active contour.

**Note 3**: The size of the Gaussian window is also important for accurate segmentation and bias field correction. In our experiment, we choose a truncated Gaussian window of size (4*k*+1)×(4*k*+1), where *k* is the greatest integer smaller than *σ*. Thus, the choice of *σ* is related to the size of the Gaussian window.

In order to facilitate the numerical simulation, we use the membership functions *u*_1_(*s*) = *H*_*ε*_(*s*) and *u*_(_ *s*) = 1−*H*_*ε*_(*s*), where $H_{\varepsilon }(s)=\frac{1}{2}(1+\frac{2}{\pi }\arctan(\frac{s}{\varepsilon }))$ is the smooth version of Heaviside function and $\frac{dH_{\varepsilon }(s)}{ds}=\frac{1}{\pi }\frac{\varepsilon }{\varepsilon^{2}+s^{2}}=\delta_{\varepsilon }(s)$. By using the gradient flows method [37], the formulation of the variation equation with the level set function of (12) can be written as

$$\begin{array}{c} \frac{\partial \phi }{\partial t}=\delta_{\varepsilon }\left(\phi \right)\left(f_{2}-f_{1}\right)+\alpha \left(\nabla^{2}\phi -div\frac{\nabla \phi }{\left|\nabla \phi \right|}\right)+\beta \delta_{\varepsilon }\left(\phi \right)\left(div\frac{\nabla \phi }{\left|\nabla \phi \right|}\right) \end{array}$$ (14)

where:

$$\begin{array}{c} f_{1}=\int K\left(y-x\right){\left|I\left(x\right)-b\left(y\right)c_{1}-d\left(y\right)\right|}^{2}dy \end{array}$$

$$\begin{array}{c} f_{2}=\int K\left(y-x\right){\left|I\left(x\right)-b\left(y\right)c_{2}-d\left(y\right)\right|}^{2}dy \end{array}$$

and *b*, *c*_*i*_ and *d* are given by (13). The corresponding initial condition and boundary condition are as follows:

$$\begin{array}{cc} \phi \left(x,0\right)=\phi_{0}\left(x\right), & x\in \Omega \\ \frac{\partial \phi }{\partial n}=0, & x\in \partial \Omega \end{array}$$ (15)

where **n** denotes the exterior normal to the image boundary ∂Ω.

We use the explicit finite difference scheme to discretize the level set Equation (14) as follows:

$$\begin{array}{c} \frac{\phi^{k+1}-\phi^{k}}{\Delta t}=\delta_{\varepsilon }\left(\phi^{k}\right)\left(f_{2}-f_{1}\right)+\alpha \left(\nabla^{2}\phi^{k}-div\frac{\nabla \phi^{k}}{|\nabla \phi^{k}|}\right)+\beta \delta_{\varepsilon }\left(\phi^{k}\right)\left(div\frac{\nabla \phi^{k}}{|\nabla \phi^{k}|}\right) \end{array}$$ (16)

where *ϕ* ^*k*^ represents the values of the level set function in the k-th iteration and Δ*t* is the time step of the evolving contours.

#### Multi-phase level set variation formulation

For the case *N* > 2, we can obtain the multi-phase level set formation similar to the case *N* = 2. The difference is that we need to use *n* level set function *ϕ*_1_, *ϕ*_2_, …, *ϕ*_*n*_ to respect *N* = 2^*n*^ regions Ω_1_, Ω_2_, …, Ω_*N*_. The corresponding membership functions *u*_*i*_ can be written as

$$\begin{array}{c} u_{i}\left(\phi_{1}\left(x\right),\phi_{2}\left(x\right),...,\phi_{n}\left(x\right)\right)=\left\{\begin{array}{cc} 1, & x\in \Omega \\ 0, & else \end{array}\right. \end{array}$$ (17)

We denote Φ = (*ϕ*_1_(**x**), *ϕ*_2_(**x**), …, *ϕ*_*n*_(**x**)) for simplicity and he membership function *u*_*i*_(*ϕ*_1_(**x**), *ϕ*_2_(**x**), …, *ϕ*_*n*_(**x**)) can be written as *u*_*i*_(Φ). We focus on the case *N* = 3 in this paper, which two level set functions *ϕ*_1_ and *ϕ*_2_ can be used to define the partitions of image domain by the membership functions *u*_1_(Φ) = *H*_*ε*_(*ϕ*_1_)*H*_*ε*_(*ϕ*_2_), *u*_2_(Φ) = *H*_*ε*_(*ϕ*_1_)(1−*H*_*ε*_(*ϕ*_2_)) and *u*_3_(Φ) = 1−*H*_*ε*_(*ϕ*_1_). Similar to the two-phase case, the final energy of the multi-phase image can be written as

$$\begin{array}{c} E^{FEF}\left(b,c,d,\Phi \right)=\int \left(\sum_{i=1}^{3}\int K_{\sigma }\left(y-x\right){\left|I\left(y\right)-b\left(x\right)c_{i}-d\left(x\right)\right|}^{2}u_{i}\left(\Phi \right)dy\right)dx+E^{R}\left(\Phi \right) \end{array}$$ (18)

where *b*, **c**, and *d* can be calculated from (13). Minimization of the energy functional in Equation (18) with respect to Φ = (*ϕ*_1_, *ϕ*_2_), we obtain the following gradient descent flow equations:

$$\begin{array}{c} \frac{\partial \phi_{1}}{\partial t}=\delta_{\varepsilon }\left(\phi_{1}\right)\left(\left(f_{2}-f_{1}\right)H_{\varepsilon }\left(\phi_{2}\right)+f_{3}-f_{2}\right)+\alpha \left(\nabla^{2}\phi_{1}-div\frac{\nabla \phi_{1}}{|\nabla \phi_{1}|}\right)+\beta \delta_{\varepsilon }\left(\phi_{1}\right)\left(div\frac{\nabla \phi_{1}}{|\nabla \phi_{1}|}\right) \end{array}$$ (19)

$$\begin{array}{c} \frac{\partial \phi_{2}}{\partial t}=\delta_{\varepsilon }\left(\phi_{2}\right)\left(\left(f_{2}-f_{1}\right)H_{\varepsilon }\left(\phi_{1}\right)\right)+\alpha \left(\nabla^{2}\phi_{2}-div\frac{\nabla \phi_{2}}{|\nabla \phi_{2}|}\right)+\beta \delta_{\varepsilon }\left(\phi_{2}\right)\left(div\frac{\nabla \phi_{2}}{|\nabla \phi_{2}|}\right) \end{array}$$ (20)

$$\begin{array}{c} f_{1}=\int K\left(y-x\right){\left|I\left(x\right)-b\left(y\right)c_{1}-d\left(y\right)\right|}^{2}dy \end{array}$$

$$\begin{array}{c} f_{2}=\int K\left(y-x\right){\left|I\left(x\right)-b\left(y\right)c_{2}-d\left(y\right)\right|}^{2}dy \end{array}$$

$$\begin{array}{c} f_{3}=\int K\left(y-x\right){\left|I\left(x\right)-b\left(y\right)c_{3}-d\left(y\right)\right|}^{2}dy \end{array}$$

The numerical algorithm can be written as the following 6 steps (take the three-phase case for example):

1. Set *k* = 1 and initialize the level set functions *ϕ*_1_ and *ϕ*_2_ to be binary functions
  $$\begin{array}{c} \phi_{1}\left(x,0\right)=\left\{\begin{array}{cc} -c_{0},\; & x\in R_{1},\\ c_{0},\; & x\notin R_{1}. \end{array}\right.\phi_{2}\left(x,0\right)=\left\{\begin{array}{cc} -c_{0},\; & x\in R_{2},\\ c_{0},\; & x\notin R_{2}. \end{array}\right. \end{array}$$
  where *c*_0_ is a positive constant, *R*_1_ and *R*_2_ are arbitrarily given regions in the image domain.
2. Initialize the bias field *b* and local difference matrix *d*.
3. Compute *b*, **c** and *d* from (13).
4. Solve the level set function *ϕ*_1_ from (19).
5. Solve the level set function *ϕ*_2_ from (20).
6. Check whether the evolution is converged. If not, set *k* = *k*+1 and return to step 3.

### Performance evaluations

In this paper, we use the jaccard similarity (JS) [17, 38], the dice similarity coefficient (DSC) [17, 39], the false positive ratio (RFP), and the false negative ratio (RFN) to compare the segmentation performances of the models quantitatively. These metrics are defined as:

$$\begin{array}{cc} JS & =\frac{N(S_{g}⋂S_{m})}{N(S_{g}⋃S_{m})},\;DSC=\frac{2N(S_{g}⋂S_{m})}{N\left(S_{g}\right)+N\left(S_{m}\right)}\\ RFP & =\frac{N(S_{g}/O)}{N(S_{g})},\;\;RFN=\frac{N(S_{m}/O)}{N(S_{m})} \end{array}$$ (21)

where *N*() represents the pixel numbers of the region. *S*_*g*_ indicates the foreground of the ground truth image and *S*_*m*_ stands for the foreground obtained by the models. *O* is the common part of *S*_*g*_ and *S*_*m*_. The closer the JS and DSC values to 1, and the RFP and RFN values to 0, the better the segmentation results.

### Statistical Analysis

Statistical analysis is performed using the statistical software MedCalc [40]. To assess the performance evaluations of segmentation quality (JS, DSC, RFP, RFN) presented in (21), the tests of statistical significance are performed using 120 simulated MR images. First, we perform the F-test [41]. If the associated (two-sided) P-value is less than the conventional 0.05, the null hypothesis is rejected and the conclusion is that the two variances do indeed differ significantly. If the P-value is low (P<0.05), the variances of the two samples cannot be assumed to be equal and it should be considered to use the t-test with a correction for unequal variances (Welch t test, [42]). The variables are expressed as Mean ± SD (standard deviations). For Welch t test, when the P-value is less than the conventional 0.05, the null hypothesis is rejected and the conclusion is that the two means do indeed differ significantly.

## Results and Discussion

In this section, the two-phase level set formulation of the proposed method is tested with synthetic and real images. All of the experiments were conducted in the MATLAB 7.14 (64bit) programming environment on a personal computer with an Intel Core 2 Duo 2.80 GHz CPU, 4 GB RAM, and Windows 7 (64bit) operating system. In our experiments, we use the following default settings of the parameters for our method unless otherwise specified: *σ* = 3 (3 ≤ *σ* ≤ 10), *ε* = 1, time step Δ*t* = 0.1, *α* = 0.1/Δ*t*, and *β* = 0.003 × 255 × 255. Most of the original images in experiments can be found at the website http://www.engr.uconn.edu/~cmli/.

**Fig 4:**
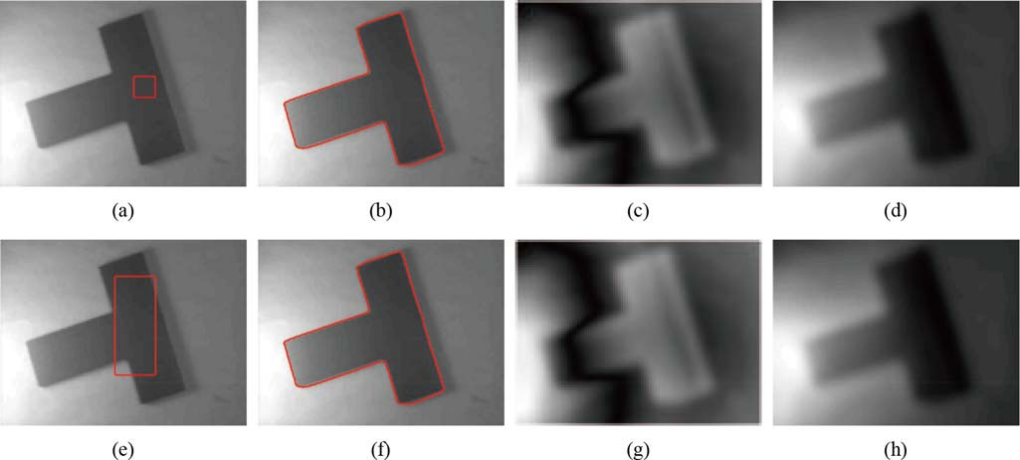
Experimental results of our model. (a), (e) The original image with red initial contours. (b), (f) The final segmentation results of our model. (c), (g) The gray images of ∣*d*∣. (d), (h) The estimation of the bias field in our model.

The next experiment considers the segmentation of the same image in Fig. 1 (as shown in Fig. 4). The T-shaped image is a real image with intensity inhomogeneity, which size is 127×96. The initial active contours are set inside the object domain and contain the background. Our method outperforms Li’s model (the code was downloaded from [44]) in some cases. As the local regional difference is considered, incorrect estimations of the true image *J* in the local region can be corrected in each iteration, which is insensitive to the initial contours in our experiments. Fig. 4 indicates that even the initial contours located inside the objects contain background, the segmentation results and bias field estimate are nearly the same. We choose the absolute value of the local difference *d*, which is shown in gray images (Fig. 4(c) and Fig. 4(g)) to describe the level between the measured image *I* and estimated *bJ*. In Figs. 4(c) and 4(g), ∣*d*∣ is often large in highlight regions or regions with similar intensities; in these regions, the difference between *I* and *bJ* must be corrected to obtain better estimations of *b* and *J*. By the quantitative comparison using the above metrics in the second row of Fig. 2 and Fig. 4, the values of JS and DSC in our method are bigger than Li’s method, the value of RFP are equal show that the region *S*_*b*_/*O* of Li’s method and our method are the same, while the value of RFN in our model (0.0068) are smaller than Li’s method (0.3186) mean the region *S*_*m*_/*O* of our method achieves more accurate segmentation results (see Table 1).

**Table 1:** JS, DSC, RFP and RFN values for the results in the second row of Fig. 2 and Fig. 4.

	*JS*	DSC	RFP	RFN
Li’s method	0.6782	0.8082	0.0047	0.3186
Our method	0.9864	0.9903	0.0047	0.0068

**Fig 5:**
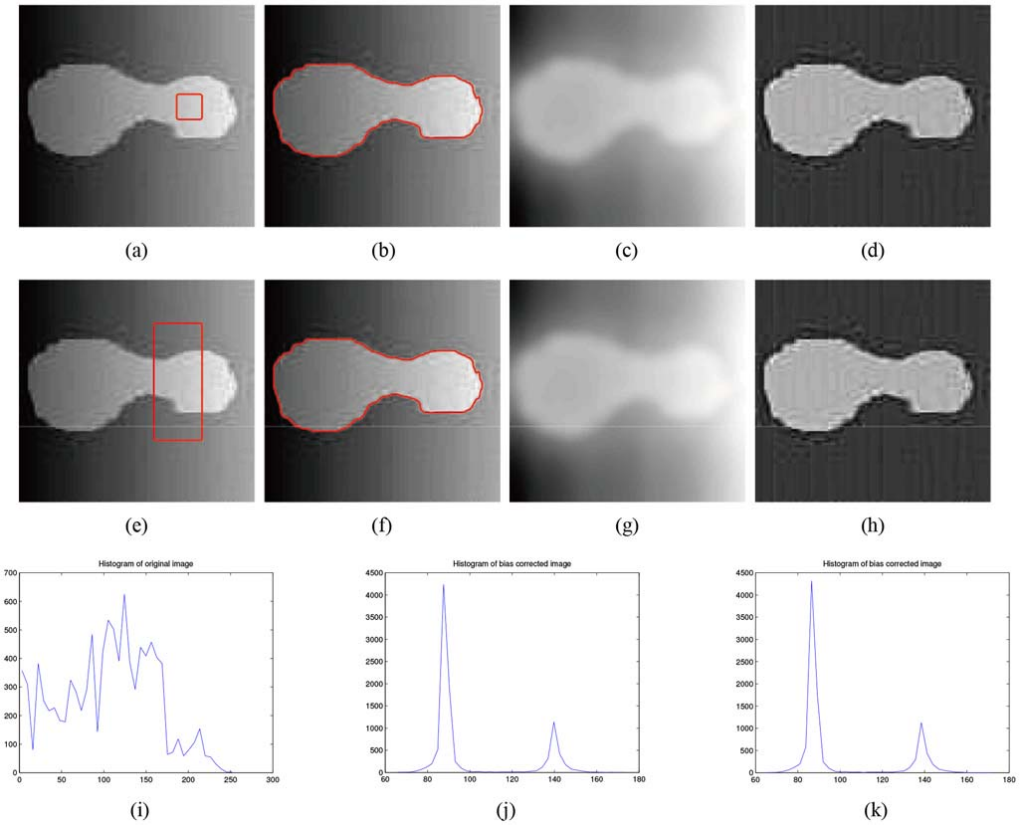
Experimental results of our model. (a), (e) The original image with red initial contours. (b), (f) The final segmentation results of our model. (c), (g) The estimation of the bias field. (d), (h) The corrected image. (i) The histogram of the original image. (j) The histogram of the corrected image with initial contour (a). (k) The histogram of the corrected image with initial contour (e).

Fig. 5 shows the segmentation results and bias field correction of the synthetic image with intensity inhomogeneity shown in Fig. 3 obtained by our model. For the distinct initial contours in Figs. 5(a) and 5(e), the corrected images are shown in Figs. 5(d) and 5(h), and the histograms of bias corrected image for different initial contours are shown in Fig. 5(j) and 5(k), which results in higher-quality image than the original image (Fig. 5(g)). The two histograms of the bias-corrected image with different initial contours are nearly identical.

**Fig 6:**
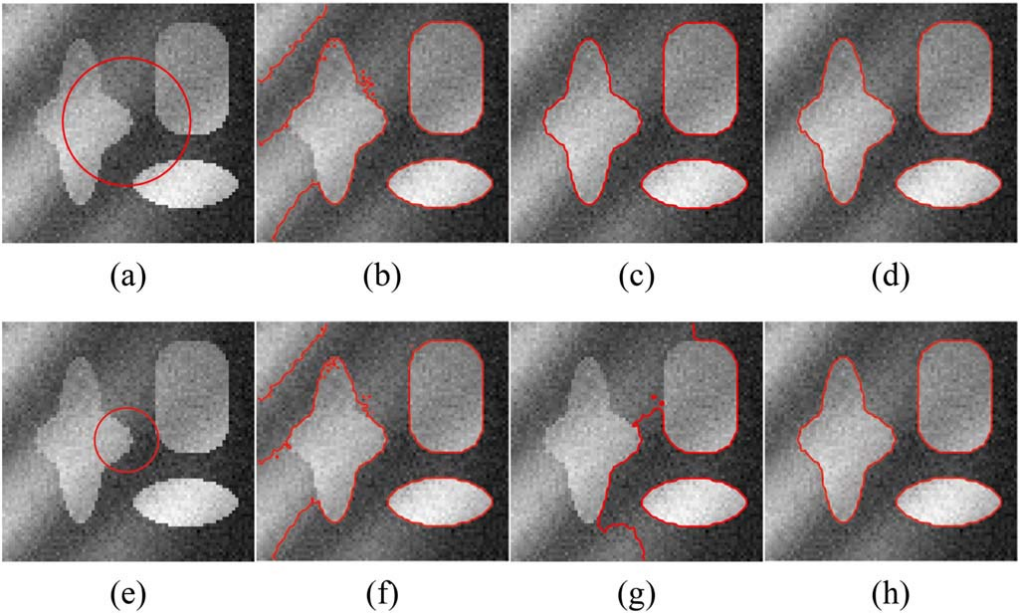
Comparisons of the segmentation results for a synthetic image with intensity inhomogeneity between Li’s model, LSACM and our model. (a), (e) The original image with red initial contours. (b), (f) The results of Li’s model. (c), (g) The results of LSACM. (d), (h) The results of our model.

**Fig 7:**
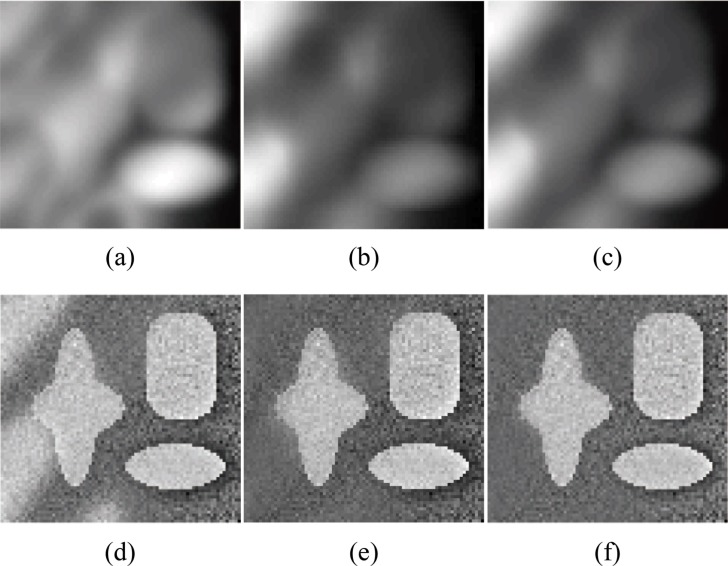
Comparisons of the bias field estimation and corrected image for Fig. 6 between Li’s model, LSACM and our model. (a), (b), (c) The estimated bias fields of Li’s model, LSACM and our model, respectively. (d), (e), (f) The corrected images of Li’s model, LSACM and our model, respectively.

Fig. 6 shows the segmentation results for a synthetic image (the image size is 79×75) with higher intensity inhomogeneity obtained with Li’s method, LSACM (code can be downloaded at [45]) and our model. In this experiment, we chose *β* = 0.007×255×255. The image contains three objects with high light on the left, and the light also causes the boundary to be fuzzy in the lower region of the star-shaped object. For Li’s method, the estimation of the true image *J* (piecewise constants) may not be accurate in the fuzzy boundary region, and thus, the estimation of the bias field may also not be accurate. The segmentation results obtained with Li’s method, LSACM and the proposed method for different initial contours are shown in columns 2, 3 and 4, respectively. Li’s method fails to segment the object boundary or estimate the fuzzy bias field with high light even when the initial contour across three objects. While LSACM can obtain the right segment result for the initial contour shown in Fig. 6 (a), but for the initial contour as Fig. 6 (e), LSACM get the undesired results even the iterate number over than 2000. The experimental results of our method are more accurate than those obtained with Li’s method and more robustness than LSACM. In Figs. 6(d) and 6(h), the final contours of our method can converge to the correct boundaries precisely. The bias field estimation and corrected image of Li’s model, LSACM and our model are shown in Fig. 7. In the highlight region of the image, the image is not well corrected by Li’s method, LSACM and our method have the similar bias field estimation based on the right segmentation.

**Fig 8:**
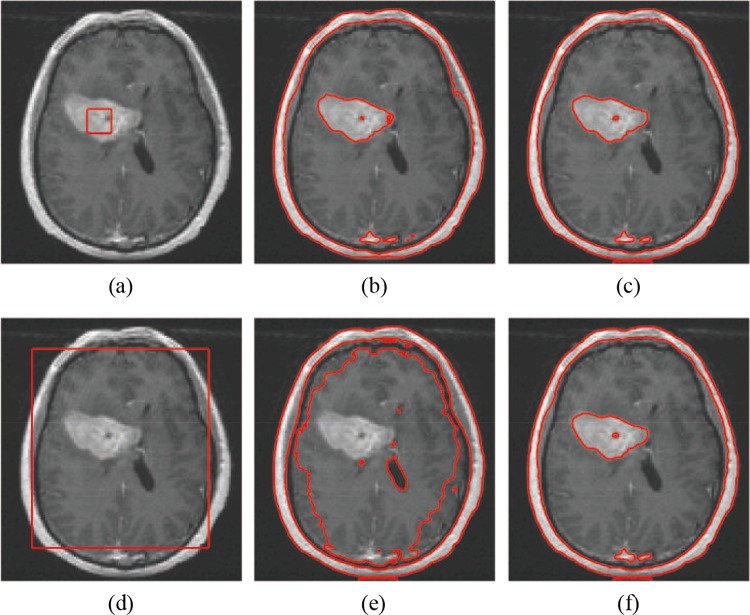
Comparisons of the segmentation results for a MR brain image contains tumor with intensity inhomogeneity between Li’s model and our model. (a), (d) The original image with initial contours. (b), (e) The results of Li’s model. (c), (f) The results of our model.

The quality of MR images is highly dependent upon the coil used to receive the RF signal emitted from the patient [43]. Fig. 8 shows the comparison between Li’s method and our method for the segmentation results with different initial contours of an MR brain image (the size is 109×119) with a tumor from the Internet. A small black spot is located at the center of the tumor. The first row shows the segmentation results with the initial contour of Fig. 8(a), and the second row shows the results with the initial contour of Fig. 8(d). Columns 1 to 3 are the original image with red initial contours, the segmentation results of Li’s method and the results of our method, respectively. Li’s method fails to acquire the tumor and spot boundaries with the initial contour shown in Fig. 8(a). The tumor boundaries obtained by Li’s model are inaccurate when the initial contour lies inside the tumor (Fig. 8(d)). Our method can obtain the boundaries of the tumor and small spot accurately because it collects the local regional difference in the entire image domain.

**Fig 9:**
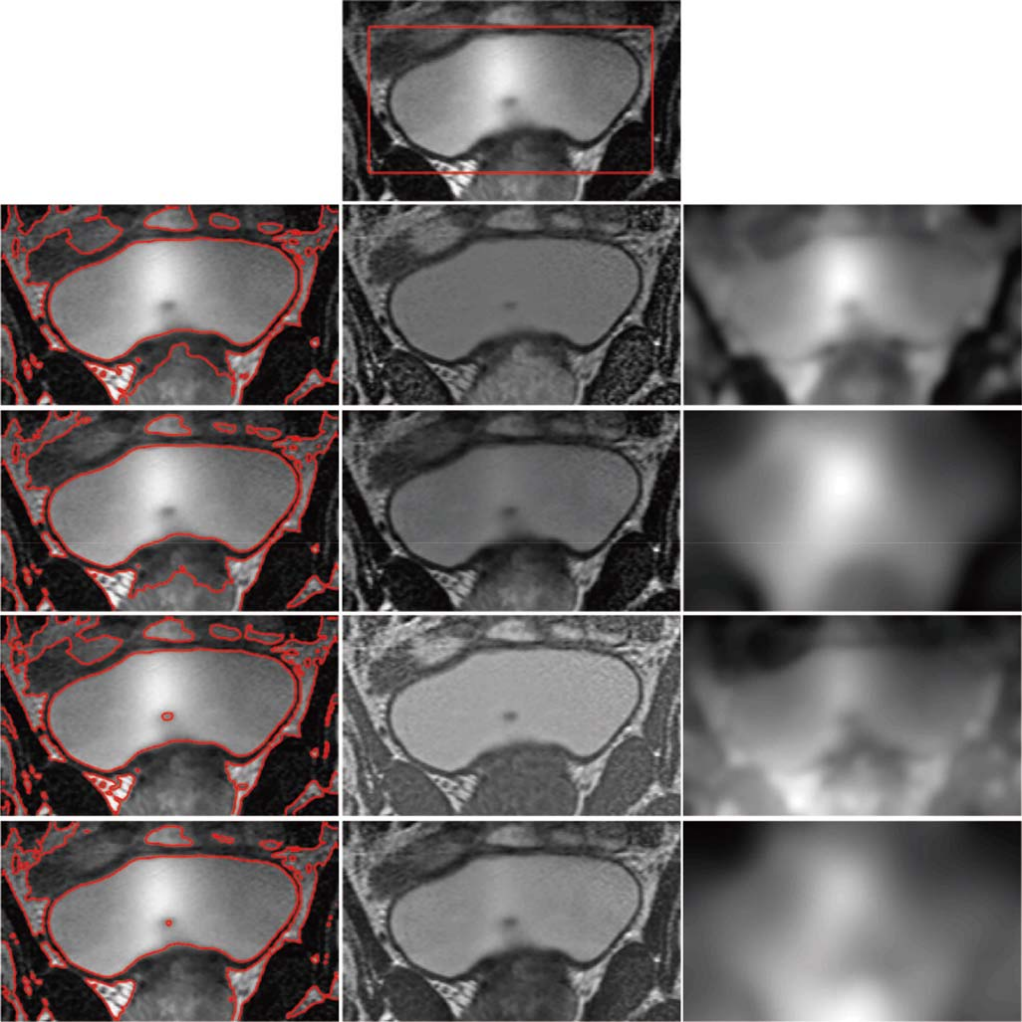
Comparisons of Li’s model and our model in different scale *σ* on MR image. Row 1 is the original image with red initial contours. The first column is the final segmentation results. The second column is the estimated bias field images. The third column is the corrected images. Row 2 and 3 are the corresponding results of Li’s model in *σ* = 3 and *σ* = 10. Row 4 and 5 are the final results of our model in *σ* = 3 and *σ* = 10 respectively.

To test the impaction of the segmentation results and corrected images for different *σ* in the Gaussian kernel function, we compare the experimental results for an MR image obtain from Li’s method and our method with different *σ*. From assumption (A1), the local region difference *d* will be more accurately collected when the size of the Gaussian window is small. Thus, in this case, more details can be considered, and the segmentation may contain more details. In contrast, choosing a larger Gaussian window size may lead to a less accurate segmentation result and increased computational. To record the computational cost for each method, we set the iteration number *n* = 200. Fig. 9 shows the comparison between Li’s method and our method with different scales *σ* = 3, 10. The original MR image with the initial contour is shown in the first row of Fig. 9, the size of the image is 180×107, and columns 1, 2 and 3 provide the final segmentation results, the estimated bias field and the corrected images, respectively. Rows 2 and 3 are the final results by Li’s method, and rows 4 and 5 are the results of our method; rows 2 and 4 consider the scale *σ* = 3, whereas rows 3 and 5 consider *σ* = 10. Fig. 9 illustrates that Li’s method fails to segment the small gray object at the center of the MR image for *σ* = 3 and *σ* = 10. Our method can accurately segment the small object in the image for the scale *σ* = 3 and segment the small object in further detail when *σ* = 10. Furthermore, the corrected images obtained with our method are superior to those obtain with Li’s method. Fig. 9 also shows that the corrected image of our method obtained when *σ* = 3 is better than that obtained when *σ* = 10; thus, the results may be more accurate for smaller *σ* than in for larger *σ*. Table 2 compares the CPU time for Li’s model and our model for different scales *σ*. The CPU time for *σ* = 10 is approximately twice that of scale *σ* = 3. Our model is more computationally burdensome than Li’s model because of the local regional difference estimation in each iteration.

**Table 2:** Comparison the CPU time of Li’s method and our method for Fig. 9.

	*σ*	CPU time(s)
Li’s method	3	11.03
	10	23.47
Our method	3	18.95
	10	40.83

**Fig 10:**
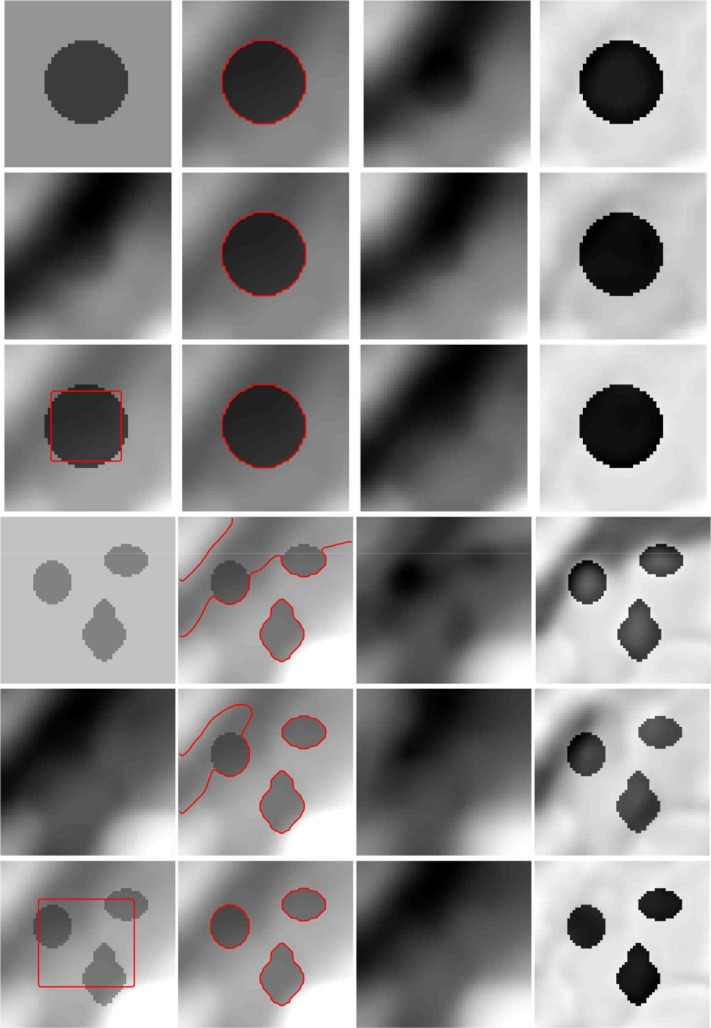
Comparisons of Li’s model, LSACM and our model for two synthesize images multiplied by given bias fields. Column 1 is the original images (row 1, 4), given bias fields (row 2, 5) and the initial contours in the images multiplied by bias fields (row 3, 6). Column 2 to 4 is the segmentation results, estimated bias fields and corrected images, respectively. Row 1 and 4 are the results of Li’s model. Row 2 and 5 are the results of LSACM. Row 3 and 6 are the results of our model.

For the observed image synthesized from the retinex model [31], we compare the performance of Li’s model, LSACM and our model for two synthesize images (which sizes are 50×50 and 64×61) multiplied by bias fields shown in Fig. 10. Column 1 in Fig. 10 shows the synthesize images (row 1 and row 4), the corresponding multiplicative bias fields (row 2 and row 5) and the initial contours for the multiplied images (row 3 and row 6), respectively. Column 2 to 4 shows the segment results, the estimated bias fields and the corrected images. Rows 1 and 4 are the results of Li’s model, rows 2 and 5 show the results of LSACM, and rows 3 and 6 are the results of our model. As shown in Fig. 10, three models can obtain similar bias fields under the premise of right segmentations (rows 1, 2 and 3). However, for the initial contour shown in row 6 (column 1), the estimated bias field of our model are more similar to the given bias field than other two models (rows 4, 5 and 6). That is to say, our model may show better performance than Li’s model and LSACM in this statement.

**Fig 11:**
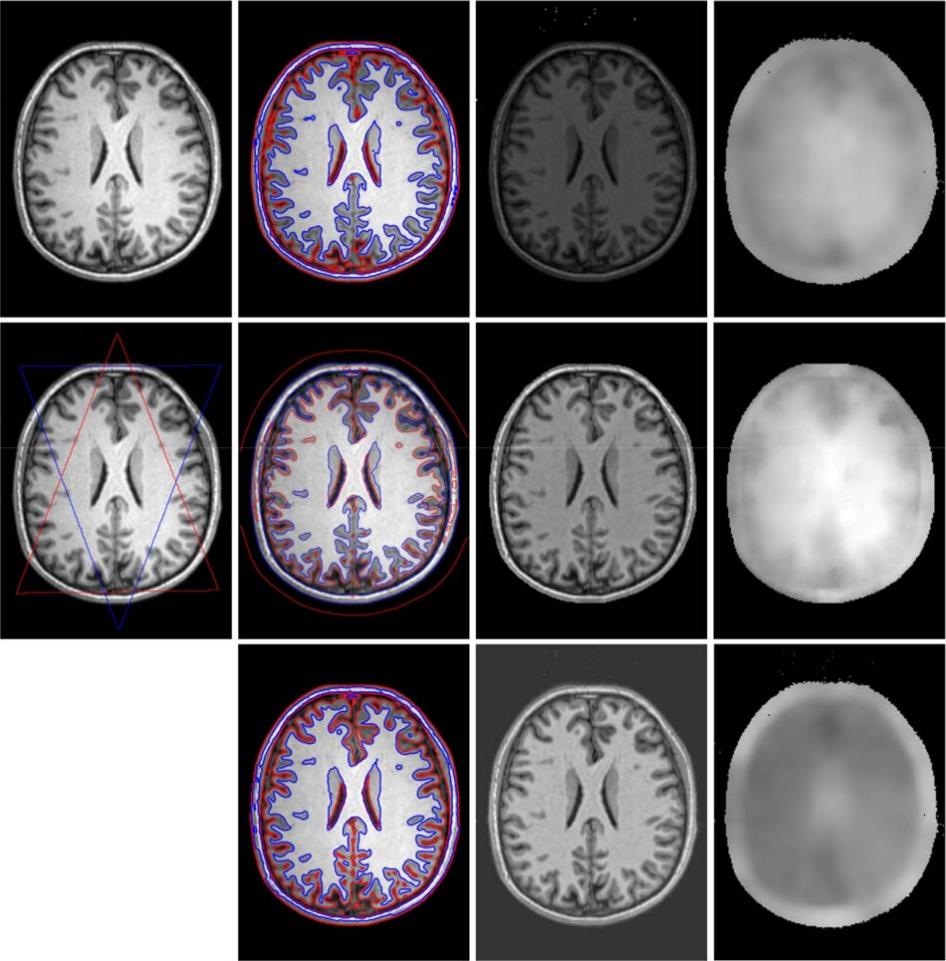
Comparisons of Li’s model, LSACM and our model for MR brain image. Column 1 is the original image with red and blue initial contours. Column 2 to 4 is the final segmentation results, the corrected images and the estimated bias field images, respectively. Row 1 to 3 is the results of Li’s model, LSACM and our model, respectively.

**Fig 12:**
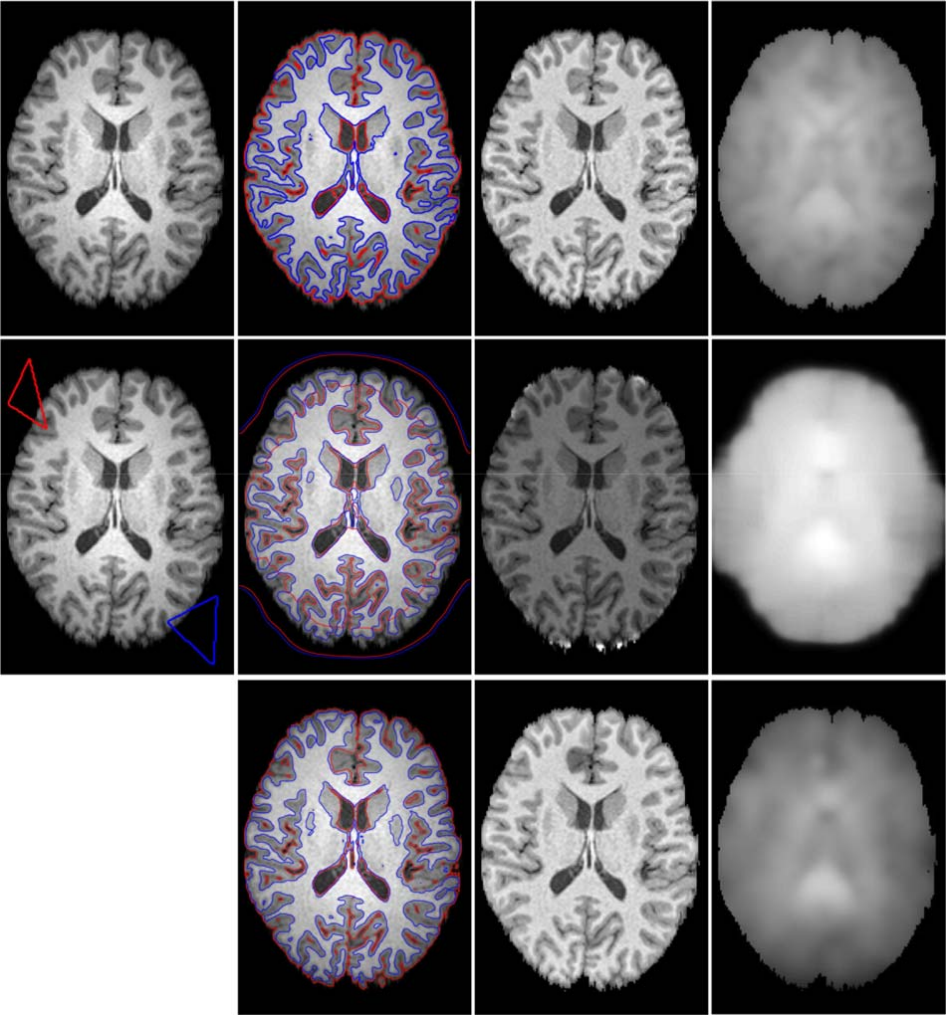
Comparisons of Li’s model, LSACM and our model for MR brain image. Column 1 is the original image with red and blue initial contours. Column 2 to 4 is the final segmentation results, the corrected images and the estimated bias field images, respectively. Row 1 to 3 is the results of Li’s model, LSACM and our model, respectively.

In the following experiments, we compare our model with Li’s model [29] and LSACM [30] (the code was downloaded from [45]) in the performance of multi-phase MR images. Fig. 11 shows the segmentation and bias-correction results on 3T MR image (from [45], the image size is 174×238), which contains white matter (WM), gray matter (GM), cerebrospinal fluid (CSF) and background (CSF usually as the background in our method). We use red contour to represent *ϕ*_1_ = 0, and blue to represent *ϕ*_2_ = 0. The first column shows the original image and initial contours, the second column shows the segmentation results, the third and fourth columns show the corrected images and bias fields, respectively. The first, second and third rows show the results of Li’s method, LSACM and our method, respectively. As we see from Fig. 11, our method and LSACM capture more CSF than Li’s method and our method obtain more accuracy of GM (WM) in the center of image than other two methods. The corrected images of LSACM and our method seem similar, which are better than Li’s method. For the brain MR image in Fig. 12, the original image (the image size is 141×202) and initial contours are shown in column 1, row 1, 2 and 3 are the results of Li’s method, LSACM and our method, respectively. LSACM can obtain the accurate boundaries of WG (blue contours), but the segmentation of CSF is unexpected (red contours), which can not be well separated the WM and GM. Li’s method can not segment the GM in the center of image. Fig. 11 and Fig. 12 show that our method have more capacity of WM and GM segmentation. The corrected images of Li’s method and our method seem similar and more vivid than LSACM.

**Fig 13:**
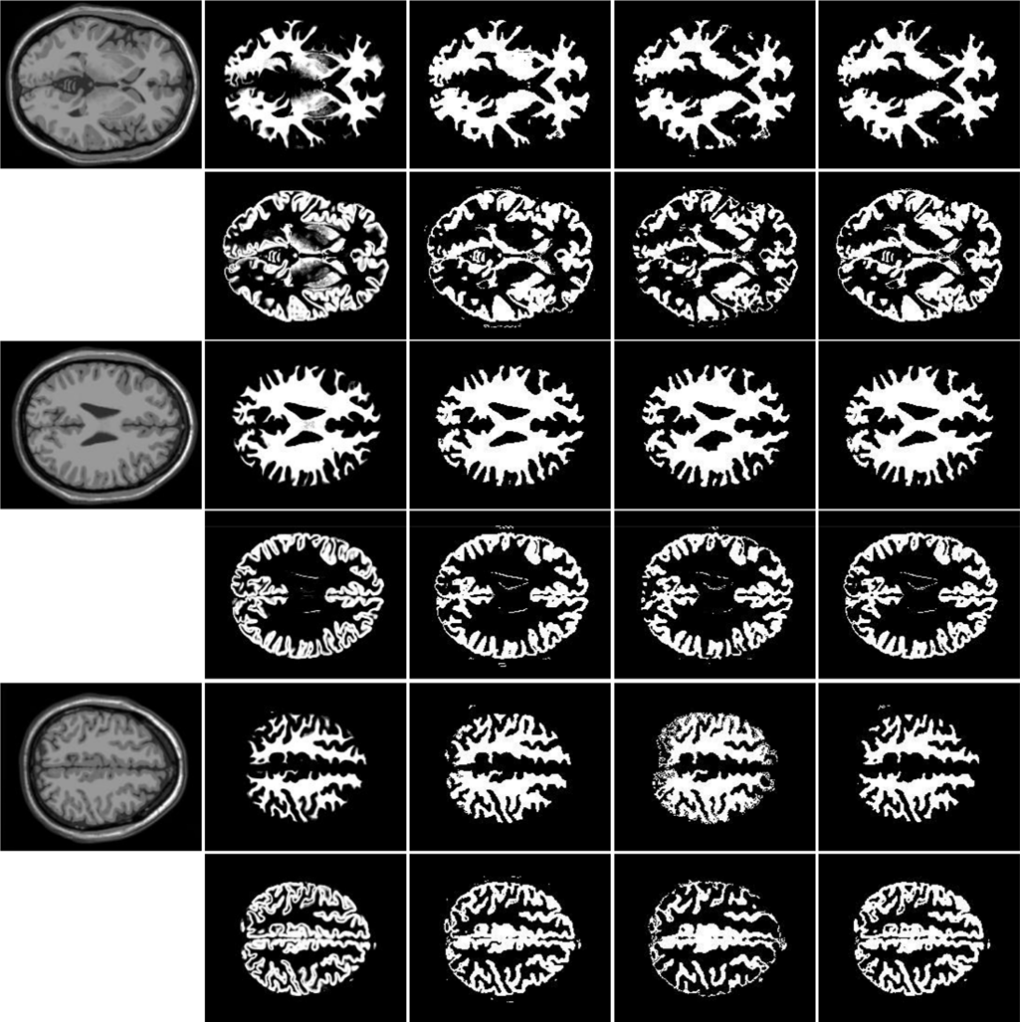
Comparisons of the segmentation results for WM and GM among Li’s model, LSACM and our model. Column 1 are the original images. Column 2 is the ground truth of original images. Column 3 to 5 is the final segmentation results of Li’s method, LSACM and our method, respectively. Rows 1, 3 and 5 are WM, and rows 2, 4 and 6 are GM. All WM and GM are displayed in white.

To further support the conclusion statements, we compare the performance of WM and GM segmentation of simulated MR image volumes for normal brain (the data can be download from [46], the image size of each slice is 217×181). We use 120 slices, which contain both WM and Gm for comparison. As shown in Fig. 13, three original images are shown in column 1, column 2 shows the ground truth of WM and GM, column 3 to 5 shows the results of Li’s method, LSACM and our method, respectively. Rows 1, 3 and 5 are WM, and rows 2, 4 and 6 are GM, all WM and GM are displayed in white. As mentioned above, we first perform the F-test. Since the P-values are lower than 0.05, the variances of these samples cannot be assumed to be equal. We need to perform the Welch’s t test. The statistical analysis results of performance evaluations of segment accuracy for WM and GM between different method are summarized in Table 3 and Table 4, respectively. The variables are expressed as Mean±SD. From Table 3, there are obvious statistical differences of JS values between Li’s method and LSACM (P<0.0001), Li’s method and our method (P = 0.0022). The values of JS for LASM (0.7146±0.0091) and our method (0.6994±0.0175) is higher than Li’s method (0.6560±0.0060). There is no obvious statistical difference in the value of JS between LSACM and our method (P = 0.3095>0.05). There are obvious statistical differences of DSC values between Li’s method and LSACM (P = 0.0001), LSACM and our method (P = 0.0003). The value of DSC for our method (0.8620±0.0019) is higher than Li’s method (0.8140±0.0141) and LSACM (0.8286±0.0081). There is no obvious statistical difference in the value of DSC between LSACM and Li’s method (P = 0.2826>0.05). There is no obvious statistical difference of RFP between Li’s method and LSACM (P = 0.0948>0.05), LSACM and our method (P = 0.6486>0.05). There is obvious statistical difference in the of RFP between Li’s method and our method (P = 0.0454<0.05). The value of RFP for our method (0.1598±0.0012) is lower than that of Li’s method (0.1714±0.0028). There is no obvious statistical difference in the value of RFN between Li’s method and LSACM (P = 0.3407>0.05). There is obvious statistical difference of RFN between Li’s method and our method (P<0.0001), LSACM and our method (P<0.0001). The value of RFN for our method (0.1130±0.0041) is lower than Li’s method (0.1876±0.0238) and LSACM (0.1710±0.0127). From Table 4, there are obvious statistical differences in the values of JS and DSC between any two methods of Li’s method, LSACM and our method (the minimized P = 0.0137). And the values of JS and DSC for our method (JS: 0.6842±0.0014, DSC: 0.8608±0.0004) are significantly higher than that of Li’s method (JS: 0.6442±0.0048, DSC: 0.7814±0.0028) and LSACM (JS: 0.6137±0.0132, DSC: 0.7534±0.0107). There is no obvious statistical differences in the value of RFP between Li’s method and LSACM (P = 0.9731>0.05), LSACM and our method (P = 0.0511>0.05). And there is obvious statistical differences in the value of RFP between Li’s method and our method (P = 0.0337). The value of RFP for our method (0.1293±0.0007) is significantly lower than that of Li’s method (0.1360±0.0005). There are obvious statistical differences in the values of RFN between any two methods of Li’s method, LSACM and our method (the minimized P = 0.0080). And the value of RFN for our method (0.1480±0.0008) is significantly lower than that of Li’s method (0.2826±0.0065) and LSACM (0.3200±0.0170).

**Table 3:** Summary of Welchs t test analysis results of performance evaluations of WM segmentation quality between different methods (for 120 simulated MR image slices of the normal brain).

	Li’s method (A)	LSACM (B)	Our method (C)	P-value		
				A vs. B	A vs. C	B vs. C
JS	0.6560±0.0060	0.7146±0.0091	0.6994 ± 0.0175	< 0.0001	0.0022	0.3095
DSC	0.8140± 0.0141	0.8286±0.0081	0.8620±0.0019	0.2826	0.0001	0.0003
RFP	0.1714± 0.0028	0.1618±0.0011	0.1598±0.0012	0.0948	0.0454	0.6486
RFN	0.1876± 0.0238	0.1710±0.0127	0.1130±0.0041	0.3407	< 0.0001	< 0.0001

**Table 4:** Summary of Welchs t test analysis results of performance evaluations of GM segmentation quality between different methods (for 120 simulated MR image slices of the normal brain).

	Li’s method (A)	LSACM (B)	Our method (C)	P-value		
				A vs. B	A vs. C	B vs. C
JS	0.6442± 0.0048	0.6137±0.0132	0.6842±0.0014	0.0137	< 0.0001	< 0.0001
DSC	0.7814± 0.0028	0.7534±0.0107	0.8608±0.0004	0.0091	< 0.0001	< 0.0001
RFP	0.1360± 0.0005	0.1359±0.0007	0.1293±0.0007	0.9731	0.0337	0.0511
RFN	0.2826± 0.0065	0.3200±0.0170	0.1481±0.0008	0.0080	< 0.0001	< 0.0001

## Conclusions

In this paper, we developed a new model for image segmentation with intensity inhomogeneity and bias field estimation. We firstly defined a local intensity clustering criterion function by considering the local difference between the measured image and estimated image. Then, the energy is minimized by a level set evolution process. A regularization is used in the level set process to ensure that the active contours are smooth and eliminate the re-initialization of level set function in the evolution of the active contours. We further extend our model into a multi-phase one to segment multi-phase images to segment WM and GM in the simulated normal brain MR image volumes. For 120 MR image slices, our method outperforms Li’s method in terms of JS, DSC, RFP and RFN for WM and GM. Our method outperforms LSACM in terms of DSC and RFN for WM, JS, DSC and RFN for GM. Our method can obtain accurate segmentation results and accurate estimations of the bias field. Experimental results on synthetic and real images demonstrate that our model is efficient.
